# Bilirubin Oxidation End Products (BOXes) Induce Neuronal Oxidative Stress Involving the Nrf2 Pathway

**DOI:** 10.1155/2021/8869908

**Published:** 2021-07-30

**Authors:** Yinzhong Lu, Wenyi Zhang, Bing Zhang, Stefan H. Heinemann, Toshinori Hoshi, Shangwei Hou, Guangming Zhang

**Affiliations:** ^1^Department of Anesthesiology, Tongren Hospital, Shanghai Jiao Tong University School of Medicine, Shanghai 200336, China; ^2^Hongqiao International Institute of Medicine, Tongren Hospital, Shanghai Jiao Tong University School of Medicine, Shanghai 200336, China; ^3^Center for Molecular Biomedicine, Department of Biophysics, Friedrich Schiller University Jena & Jena University Hospital, Hans-Knöll-Str. 2, D-07745 Jena, Germany; ^4^Department of Physiology, University of Pennsylvania, Philadelphia, PA 19104, USA

## Abstract

Delayed ischemic neurological deficit (DIND) is a severe complication after subarachnoid hemorrhage (SAH). Previous studies have suggested that bilirubin oxidation end products (BOXes) are probably associated with the DIND after SAH, but there is a lack of direct evidence yet even on cellular levels. In the present study, we aim to explore the potential role of BOXes and the involved mechanisms in neuronal function. We synthesized high-purity (>97%) BOX A and BOX B isomers. The pharmacokinetics showed they are permeable to the blood-brain barrier. Exposure of a moderate concentration (10 or 30 *μ*M) of BOX A or BOX B to isolated primary cortical neurons increased the production of reactive oxygen species. In the human neuroblastoma SH-SY5Y cells, BOX A and BOX B decreased the mitochondrial membrane potential and enhanced nuclear accumulation of the protein Nrf2 implicated in oxidative injury repair. In addition, both chemicals increased the mRNA and protein expression levels of multiple antioxidant response genes including *Hmox1*, *Gsta3*, *Blvrb*, *Gclm*, and *Srxn1*, indicating that the antioxidant response element (ARE) transcriptional cascade driven by Nrf2 is activated. In conclusion, we demonstrated that primary cortical neurons and neuroblastoma cells undergo an adaptive response against BOX A- and BOX B-mediated oxidative stress by activation of multiple antioxidant responses, in part through the Nrf2 pathway, which provides in-depth insights into the pathophysiological mechanism of DIND after SAH or other neurological dysfunctions related to cerebral hemorrhage.

## 1. Introduction

Subarachnoid hemorrhage (SAH) is a serious cerebrovascular complication with complex underlying mechanisms inflicting brain perfusion and function. Despite great progress in the understanding of SAH pathophysiology and management of ruptured aneurysms, SAH remains a severe and significant health problem [1]. SAH causes early brain injury, which may be followed after 7 to 14 days by delayed ischemic neurological deficit (DIND). DIND is the leading cause of morbidity and mortality in the patients who survive the initial impact of SAH and have had their aneurysm effectively treated [2]. However, the etiology and pathophysiology of SAH and DNID remain incompletely understood.

Accumulating evidence has shown that oxidative stress, acute and subsequent consisting immunological response, and other factors collectively cause the severe adverse effects of SAH [3, 4]. Erythrocyte lysis, a major initiator, is the release of heme and its subsequent degradation cascade, which collectively exert a strong effect on the affected neurons and their electrical excitability [5–7]. Various heme and heme degradation products (HHDPs) were identified in the cerebrospinal fluid (CSF) from the patients after SAH or other types of brain hemorrhage [8–11] and serum from hepatic failure patients [12].

Notably, an array of bilirubin oxidation end products (BOXes) with small molecular weight were identified [11, 13, 14] and detected in the CSF of a patient with DIND after SAH [8, 9, 15]. Several studies using crude extraction or synthesized BOXes have shown that BOXes constrict mouse cortical blood vessels and damage the normal contractile of vascular smooth-muscle cells [16, 17], suggesting that BOXes are involved in the delayed cerebral vasospasm, a potential cause of DIND. Our previous study showed that a crude BOXes mixture regulates the blood tone by modulation of Ca^2+^- and voltage-gated K^+^ channels (Slo BK_Ca_) [18]. However, the direct impact of BOXes on neuronal cells remains to be examined.

In this study, we hypothesized that BOX A or BOX B are important functional regulators of neurons during the progression of DIND after SAH. Therefore, we synthesized BOX A and BOX B following the reported protocol [14, 19] and examined their biological effects on cultured primary cortical neurons and neuronal cell lines to elucidate the underlying molecular mechanisms.

## 2. Materials and Methods

### 2.1. Reagent and Antibodies

2′,7′-Dichlorofluorescein diacetate (DCF-DA) and dimethyl sulfoxide (DMSO) were purchased from Sigma-Aldrich, the antibody raised against HO-1 (1 : 500), BLVRB (1 : 500), and Gclm (1 : 1000) from Proteintech Group, Inc. (Chicago, IL, USA); Nrf2 (WB, 1 : 1000, IF, 1 : 200) and Gsta3 (1 : 500) from Abcam (Cambridge, MA, USA); SRXN1 (1 : 500) from Bioss (Beijing, China); Caspase 3 (WB, 1 : 1000) from Abmart (Shanghai, China); and *β*-actin (WB, 1 : 2000) from OriGene Technologies, Inc. (Rockville, MD, USA). Alexa Fluor 546 anti-mouse secondary Ab (IF:1 : 2000, A-11030), goat anti-mouse IgG (H + L) peroxidase conjugated (WB: 1 : 20000, 31430), and goat anti-rabbit IgG (H + L) peroxidase conjugated (WB: 1 : 20000, 31460) were purchased from Thermo Fisher Scientific (Waltham, MA, USA).

### 2.2. BOXes Synthesis and B/P Study

**Figure 1:**
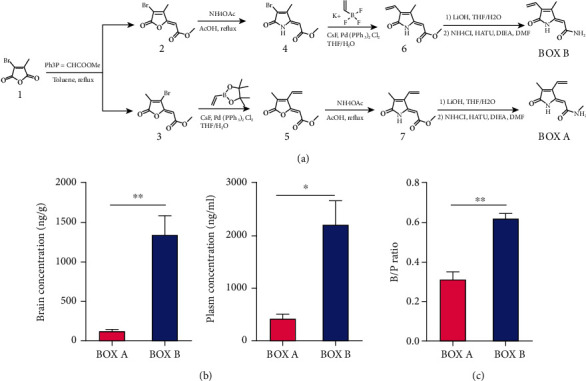
Synthesis of BOX A and BOX B and their permeability though the blood-brain barrier. (a) The chemical synthesis scheme of BOX A and BOX B. (b) Brain concentration (ng/g) and plasma concentration (ng/mL) of male ICR mice receiving in vein injection with a single dose of 12 mg/kg BOX A or BOX B and 30 min later, then collected samples for measurement. (c) Ratio of brain concentration to the plasm concentration (B/P ratio) of BOX A and BOX B in ICR mice. (b and c) Data represent Mean ± SEM (*n* = 3), unpaired Student's *t*-test, ^∗^*P* < 0.05, and ^∗∗^*P* < 0.01.

BOX A and BOX B were synthesized as shown in Figure 1(a). Compound 1 (bromocitraconic anhydride) reacts with triphenylphosphoylide in toluene with reflux. Because the two carbonyl reaction sites are different, two products can be obtained: 3 is the primary product, 2 is the by-product, and two of which can be separated by silica gel column chromatography. Compounds 2 and 3 have the reactivity of palladium-catalyzed coupling reaction and amine acetate substitution reaction (the reaction order is not required). Then, compounds 6 and 7 were hydrolyzed into carboxylic acid intermediates by alkali hydrolysis, and then the target products BOX A and BOX B were obtained by an amino substitution reaction with ammonium chloride. The target products are purified by HPLC and identified by LC/MS/MS. BOX A or BOX B were measured by a validated HPLC-MS/MS method. The assay used an automated system followed by HPLC (Shimadzu LC30AD) using a Waters XSELECT UPLC HSS T3 2.5 *μ*m 2.1∗50 mm column with gradient elution using mobile phase A containing 0.1% formic acid in water and mobile phase B (0.1% formic acid in acetonitrile) at a flow rate of 500 *μ*L/min. The gradient run was as follows: 10% solvent B (0.01–0.30 min), from 10% to 98% B (0.30–1.30 min), 98% B (1.30–1.80 min), from 98%B to 10% B (1.80–1.90 min), and 10%B (1.90–2.40 min). Detection was performed by MS/MS using an API4000 electrospray ionization (ESI) mass spectrophotometer (Applied Biosystems, API 4000 triple quadrupole mass spectrometer, AB SCIEX, Concord, Ontario, Canada). The masses for tolbutamide were precursor ion m/z 271.2 and product ion m/z 155.1, for BOX A, precursor ion m/z 179.2 and product ion m/z 162.2, and for BOX B, precursor ion m/z 179.2 and product ion m/z 162.3.

For pharmacological kinetics study, a single dose of 12 mg/kg body weight BOX A or BOX B (powder dissolved in 5% DMSO : 95% saline (pH = 11) [20, 21] with final concentration 2.4 mg/mL) was injected *in vein* (*i.v.*) with 5 mL/kg body weight in male ICR mice (*n* = 3) obtained from Beijing Vital River Laboratory Animal Technology Co., Ltd. (Beijing, China). After 30 min, the plasma concentrations (ng/mL) and the brain concentrations (ng/g) were measured. For preparations of mouse plasma and brain samples, 100 *μ*L calibration curve samples in single, QC samples in duplicate, and mouse plasma samples were mixed with 300 *μ*L acetonitrile containing Internal standard (50 ng/mL of propranolol, 200 ng/mL of tolbutamide, and 50 ng/mL of diclofenac) in Eppendorf tubes. After the mixture was vortexed for 1 min, then centrifuged for 10 min, transfer 50 *μ*L supernatant to a 96- well plate with 100 *μ*L pure water, shaking for 10 min, and finally inject 10 *μ*L into LC-MS/MS system. For brain samples, brain samples were added with 5 folds (*w*/*v*) phosphate buffer (100 mM, pH 7.4) in terms of the weight of samples, then homogenated. Subsequent procedures were performed as described for plasma. The brain concentration to the plasma concentration was calculated as B/P value. The animal study including the use of embryos for primary neuron cultures was carried out in accordance with regulations for animal experimentation and were approved by the Animal Committee of Tongren Hospital affiliated to Shanghai Jiao Tong University School of Medicine.

For other assays in this study, the ready-for-use BOX A or BOX B solution was prepared as 1,000 times in DMSO as the final concentration in administered cells and stored at -20°C in the dark. Therefore, the final concentration of DMSO in all treatments was 0.1%.

### 2.3. Primary Cortical Neurons and Neuroblastoma Cell Lines

Mouse primary cortical neurons (PCNs) were cultured as previously described [22] with minor modifications. Briefly, E17.5 ~ E18.5 embryos of either sex were dissected from pregnant C57BL/6J mice for cortex dissection in cold Dulbecco's Modified Eagle Medium (DMEM). The dissected cortices were digested with ~0.25% trypsin solution (Gibco) for ~12 min and then neutralized by DMEM containing 10% fetal bovine serum (FBS, Gibco) and agitated to make single suspension by pipetting up and down with cutting round tips. The cell suspension was pelleted by centrifugation at 1000 rpm, resuspended in complete medium, and passed through a 70 *μ*m cell strainer to exclude cell debris and aggregates. Cells were seeded at a density of 5∗10^4^/cm^2^ in culture plates or dishes that precoated with 40 *μ*g/mL poly-D-lysine (Sigma-Aldrich) and regarded as 0 days *in vitro* (DIV). About 2 ~ 4 h after plating, the cell culture medium was replaced with neural basal medium containing 1% penicillin/streptomycin, 1% Glutamax™, and 2% B27 supplement (Gibco). Subsequently, the medium was half-changed every 3 days till PCNs developing at 8 ~ 11 DIV for the compound treatment or analysis.

Human neuroblastoma SH-SY5Y and mouse neuroblastoma Neuro2a cells were obtained from the Shanghai Cell Bank of the Chinese Academy of Science (Shanghai, China) and maintained in DMEM containing 10% FBS and 1% penicillin/streptomycin in appropriate culture plates.

### 2.4. Cell Viability Assay and TUNEL Assay

Cell viability was determined by using a Cell Counting Kit-8 (CCK-8; DOJINDO) to test the impact of BOX A and BOX B according to the manufacturer's instructions. Briefly, the CCK-8 (10 *μ*L/well) was added to 96-well plate culture dishes and incubated for 2 h for detection by the multifunctional microplate detector (BioTek). Data was interpreted as the percentage of control (vehicle).

For the TUNEL assay, the BOX A- or BOX B-treated cortical neurons were fixed in 4% paraformaldehyde and detected by *In Situ* Cell Death Detection Kit, Fluorescein (Roche). Before mounting, cells were counterstained with Hoechst33342 (DOJINDO, Japan). The apoptosis rate was calculated as the percentage of TUNEL positive cells to the Hoechst33342 stained cells.

### 2.5. ROS Detection

DCF-DA (10 *μ*M) was added to the BOX A- or BOX B- or vehicle-treated cells in multiplate incubating for 30 min, subsequently the free probes was washed outandcells were further incubated for 30 min for readouts by using the fluorescence filter (Ex/Em: 488/525). The arbitrary fluorescence intensity was interpreted as the percentage of vehicle-treated group cells. For the ROS detection by imaging, similar incubation with DCF-DA was mentioned above and photographed by fluorescence microscopy (Olympus, Japan).

### 2.6. Mitochondrial Membrane Potential Measurement

Briefly, SH-SY5Y cells were seeded to grow on 6-well culture plate 24 hr prior to the indicated concentrations of BOX A or BOX B. 48 hr later, mitochondrial membrane potential measurements was carried out with the BD™ MitoScreen (JC-1) Kit (Cat. No. 551302, BD Pharmingen™) according to manufacturer's instruction. The results were analyzed by using BD Accuri C6 Software (TreeStar, San Carlos, CA, USA).

### 2.7. RNA Extraction and Agilent Microarray

Total RNA was extracted by Trizol reagent (Life-technology, USA) and purified by RNeasy Mini Kit (Qiagen#74106). The RNA passed the quality control by Agilent 2100 bioanalyzer (RIN > 6.0 and 28S/18S ≥ 0.7) could be qualified to subsequent Agilent SurePrint G3 Mouse Gene Expression 8 × 60 K. The original array figures were subjected to data normalization. After normalization, fold change (multiple of expression difference) and Student's *t*-test were used for screening and significance statistics (Sinotech Genomics, Shanghai, China).

### 2.8. Reverse Transcription and qPCR

RNA isolation and RT-qPCR were performed as previously described [23]. Briefly, RNA was extracted by Trizol reagent, 1 *μ*g total RNA was reversed transcribed by ReverTra Ace kit (TOYOBO), and the cDNA was used to quantify by the lightcycle480 system (Roche) with 2 × Power SYBRgreen mastermix (Applied Biosystems, Carlsbad, CA, USA). Quantification of gene expression was calculated by normalization to GAPDH using the 2^-*ΔΔ*Ct^ method. Primer sequences for qPCR could be available on request.

### 2.9. Protein Extraction, Western Blotting, and Quantification

As described previously [23], cells were lysed in Glo Lysis Buffer (Promega) supplemented with protease inhibitor and phosphatase inhibitor cocktails (Roche). Next, 20–60 *μ*g of total proteins (as determined by BCA assay kit) was resolved by 10% or 12% SDS-PAGE gel, transferred onto a 0.2-*μ*m PVDF membrane (Millipore, CA, USA), and probed with antibodies as indicated in the figures.

### 2.10. Immunofluorescence Staining

Immunofluorescence staining was performed as in our previous study [23]. Briefly, cells were fixed in 4% PFA, washed with PBS, permeabilized in 0.02% Triton X-100, blocked with 1% bovine serum albumin for 1 h at room temperature, and incubated with Nrf2 antibody (1 : 1000, Cell Signaling Technologies) overnight at 4°C. Cells were then incubated with Alexa Fluor^®^546 conjugated goat anti-rabbit IgG (H + L) (1 : 1000, Invitrogen) and counterstained with Hoechst33342 (DOJINDO). The stained cells were photographed with A1 scope microscope (Zeiss, Germany).

### 2.11. Data Analysis and Statistics

All figures photographed or cropped were assembled by using the Adobe illustrator CS5 software. All quantitative data was expressed as a percentage of control or vehicle (mock). Statistical analysis was performed on raw data for each group by Student's *t*-test or ANOVA with multiple comparisons post hoc analysis as indicated in the respective figure legends by GraphPad Prism 8. The resulting *P* values are provided solely as data descriptors without any inferential intent.

## 3. Results

### 3.1. BOX A and BOX B Are Permeable to the Blood-Brain Barrier

To address the physiological and pathophysiological role of BOXes in brain neurons, we synthetized BOX A and BOX B (Figure 1(a) and Supplemental Figure 1) with a predicted mass ~180 Da, which is consistent with the previous reports [14, 19]. HPLC separation and subsequent UV-vis spectroscopic measurement verified a purity of more than 97%. Pharmacokinetics studies in ICR male mice showed a moderate distribution in brain and plasma (Figure 1(b)), and the calculated brain/plasma (B/P) ratio showed a more pronounced brain enrichment for BOX B (Figure 1(c)). These results suggested that BOX A or BOX B derived from the ruptured cerebral artery are permeable through the blood-brain barrier and have the potential to affect neuronal function.

### 3.2. BOX A and BOX B Do Not Affect Cell Viability or Apoptosis in Cortical Neurons

**Figure 2:**
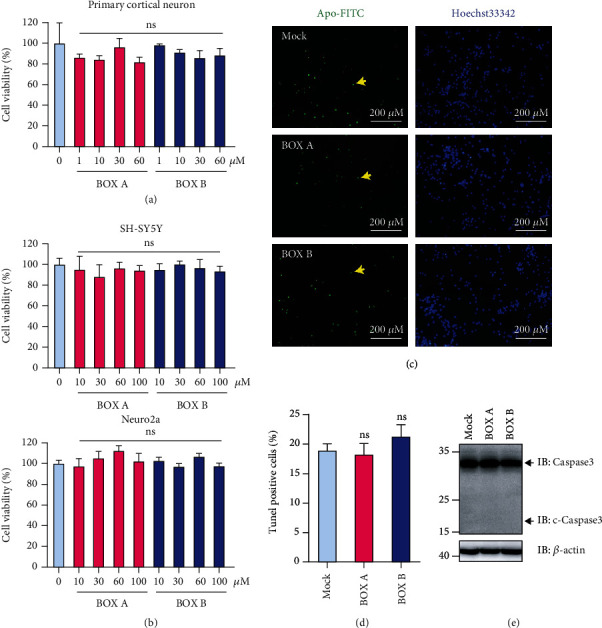
BOX A and BOX B do not affect cell viability and apoptosis. (a and b) Relative viability of primary cortical neurons (PCNs, *n* = 3) cultured 11 days in vitro (DIV) (a) and neuroblastoma cells (top, SH-SY5Y, *n* = 5; bottom, Neuro2a, *n* = 6) (b), for the indicated concentrations of BOX A (red) and BOX B (blue). Vehicle (0.1% DMSO) was applied in the control experiments (pale blue). (c) A representative TUNEL staining of 13 DIV PCNs treated with BOX A or BOX B (30 *μ*M) for 72 h. The apoptotic PCNs were stained with Apo-FITC (green) and counterstained with Hoechst33342 (blue). Bar: 200 *μ*m. (d) The statistical analysis of the calculated apoptosis (%) of the PCNs as shown in (c). The ratio of green labeled cells to the blue ones was taken as a measure for apoptosis (%) in 7 ~ 9 random photographed field. Data were presented as Mean ± SEM (*n* = 7 ~ 9), ANOVA with post Bonferroni's test. ns: no significant. (e) Representative immunoblots of caspase 3 of 11 DIV cultured PCNs treated with BOX A or BOX B (30 *μ*M each) for 48 h. One representative blot of 3 independent experiments. The antibody against caspase 3 (#T40044, Abmart Inc.) used here could detect both caspase 3 and cleaved caspase 3 (c-Caspase3).

Given the cytotoxicity in hepatocytes treated with high doses of BOX A and BOX B [12], we employed the Cell Counting Kit-8 (CCK-8) assay to monitor the impact of BOXes on neuroblastoma cells and primary cortical neurons. Regarding cell viability, there was no obvious difference between vehicle and BOXes-treated (1 ~60 *μ*M) in cortical neuron cells (Figure 2(a)). Similar results were obtained for SH-SY5Y and Neuro2a (Figure 2(b)). In addition, BOX A or BOX B at 30 *μ*M had no apparent impact on cell apoptosis for ~48 hr, evaluated with a TUNEL assay (Figures 2(c) and 2(d)) and with a Cleaved Caspase 3 (Figure 2(e)). These results showed no effects on the cell viability and apoptosis in cortical neurons.

### 3.3. BOX A and BOX B Induce Oxidative Stress in Primary Cortical Neurons

**Figure 3:**
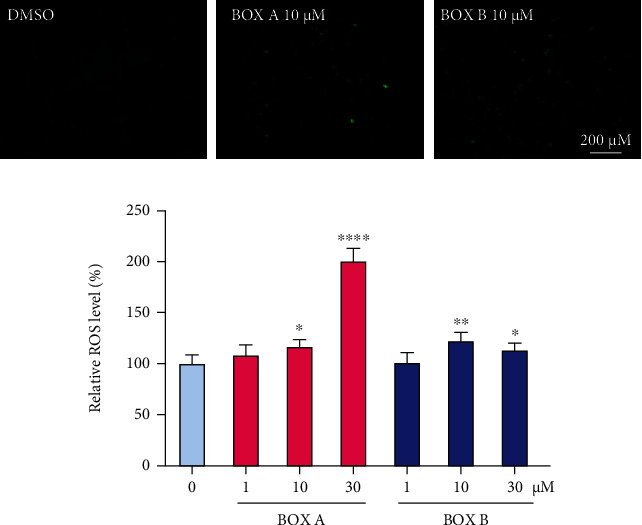
BOX A and BOX B increase the level of reactive oxygen species in primary cortical neurons. (a) A representative of reactive oxygen species (ROS) staining is shown by fluorescence imaging with DCF-DA (green). 10 DIV cultured PCNs were treated with BOX A or BOX B (10 *μ*M) for 48 h and then incubated with DCF-DA probe, washed for imaging. Bar: 200 *μ*m. (b) Normalized ROS level in PCNs treated with indicated concentrations of BOX A (red) or BOX B (blue) for 48 h. Data represented as Mean ± SEM (*n* = 4), ANOVA, and Fisher's LSD post hoc test. ^∗^*P* < 0.05, ^∗∗^*P* < 0.01, and ^∗∗∗∗^*P* < 0.0001 vs. vehicle (0.1% DMSO).

Studies in hepatocytes have shown that BOXes impact on the GSH level [12], indicating its role in redox regulations. To study whether BOX A and BOX B can cause neuronal oxidative stress, 2′,7′-dichlorofluorescein diacetate (DCF-DA), a fluorescence indicator of oxidant generation, was employed to detect reactive oxygen species (ROS) in BOXes-treated primary cortical neurons. The fluorescence showed that both BOX A and BOX B increased DCF-DA fluorescence intensity more than vehicle applications (Figure 3(a)). Similar results were obtained in DCF-DA-labeled primary cortical neurons using a multiplate reader (Figure 3(b)). These results thus suggested a more pronounced ROS production in the presence of BOX A and BOX B.

### 3.4. BOX A and BOX B Lower the Mitochondrial Potential in SH-SY5Y Cells

**Figure 4:**
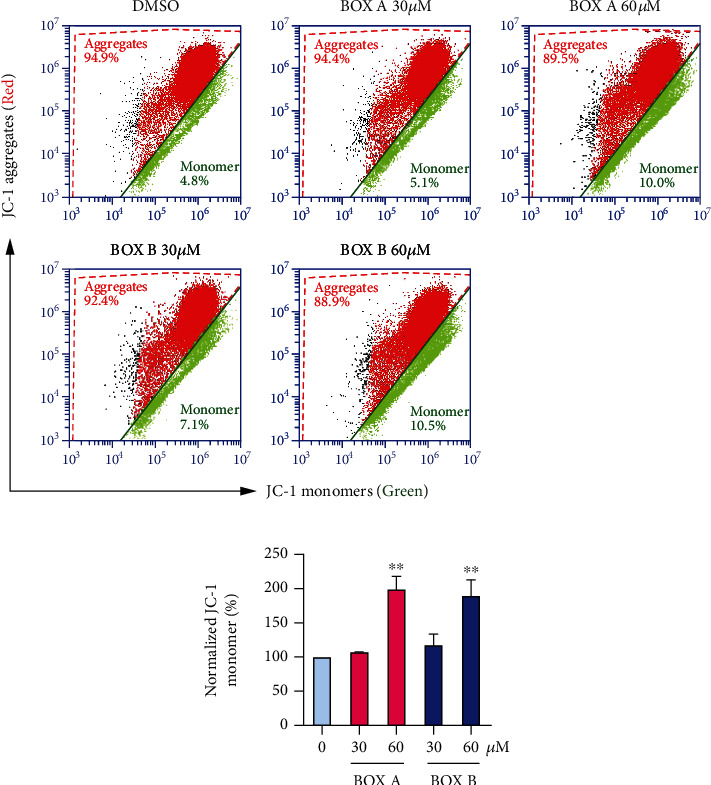
BOX A and BOX B decrease the mitochondrial membrane potential in neurons. (a) Representative FACS analysis of the mitochondrial membrane potential in SH-SY5Y cells using JC-1 staining (*n* = 3); the fraction of monomer is indicative of depolarized mitochondria. The concentrations of BOXes are indicated for 48 h incubation. (b) Normalized monomer (%) as shown in (a). Data represented as Mean ± SD (*n* = 3), ANOVA, and Bonferroni post hoc test. ^∗∗^*P* < 0.01 vs. vehicle (0.1% DMSO).

The impact of BOXes on the cellular ROS level stimulated the question if mitochondrial functions might be involved. We therefore measured the mitochondrial potential with the indicator JC-1 with and without application of BOXes. In a flow-cytometry assay to detect the percentage of the aggregated and monomer JC-1 probes, SH-SY5Y cells were treated with BOX A or BOX B. Both BOX A and BOX B increased the fraction of monomer probes (green channel) in a concentration-dependent manner (Figures 4(a) and 4(b)).

### 3.5. BOX A and BOX B Activate the Nrf2 Pathway

**Figure 5:**
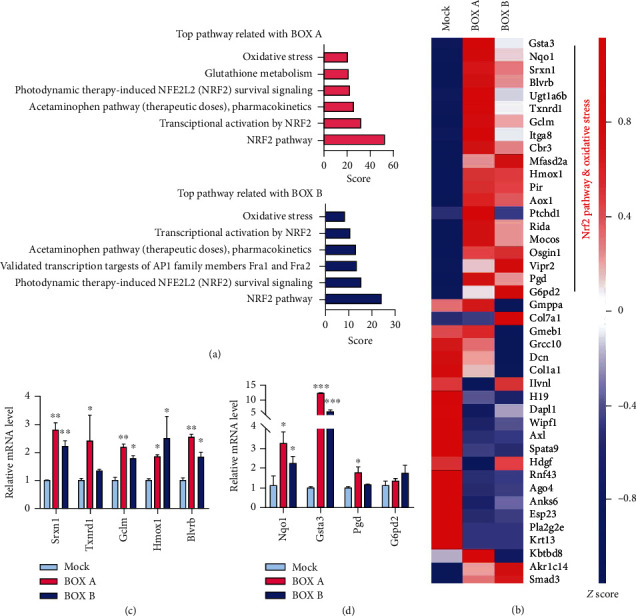
BOX A or BOX B trigger antioxidant responsive gene expression. (a) Top pathways analysis of BOX A or BOX B-treated PCNs showing the differentially expressed genes (DEGs) by microarray screening with criteria ∣fold change | >2 and *P* value < 0.05 by using online tool GeneAnalytics (http://geneanalytics.genecards.org). (b) Heatmap analysis of significantly altered DEGs (see the supplemental Table 1). (c and d) RT-qPCR validation of the DEGs screened by microarray. Fold changes were interpreted as Mean ± SEM (*n* = 3 triplicates, one representative of 3 independent experiments), ANOVA with post hoc test. ^∗^*P* < 0.05, ^∗∗^*P* < 0.01, ^∗∗∗^*P* < 0.001, compared to Mock (0.1% DMSO). (b–d) Genes are abbreviated: *Nrf2*: NF-E2 related factor 2; *Hmox1*: heme oxygenase 1; *Nqo1*: NADPH quinone oxidoreductase 1; *Blvrb*: biliverdin reductase B (flavin reductase (NADPH)); *Gclm*: glutamate-cysteine ligase, modifier subunit; *Gsta3*: glutathione S-transferase, alpha 3; *Srxn1*: sulfiredoxin 1 homolog (*S. cerevisiae*).

To elucidate the molecular mechanism underlying the BOX A- or BOX B-induced oxidative stress in neurons, a high-throughput gene expression array was used to screen differentially expressed genes (DEGs) between the BOXes- and vehicle-treated cells (Supplemental Table 1). The Gene Ontology (GO) analysis of DEGs showed that expression of those genes related to oxidoreductase activity, oxidation-reduction process, oxidative stress, and cellular response to hydrogen peroxide was greater in the BOX A- or BOX B-treated cells (Supplemental Figure 2). In addition, the pathway analysis used DEGs through GeneAnalytics (http://geneanalytics.genecards.org) showed the Nrf2-related pathway and oxidative stress were highly overpresented in either BOX A or BOX B (30 *μ*M each) treated-primary cortical neurons (Figure 5(a)). Furthermore, a subset of DEGs involved by Nrf2 and oxidative stress were clustered as shown in the heat map analysis (Figure 5(b)). Subsequently, RT-qPCR analysis further confirmed the expression changes of several aforementioned genes (Figure 5(d)).

### 3.6. BOXes Promote Nrf2 Nuclear Translocation and Protein Expression of Antioxidant Responsive Genes

**Figure 6:**
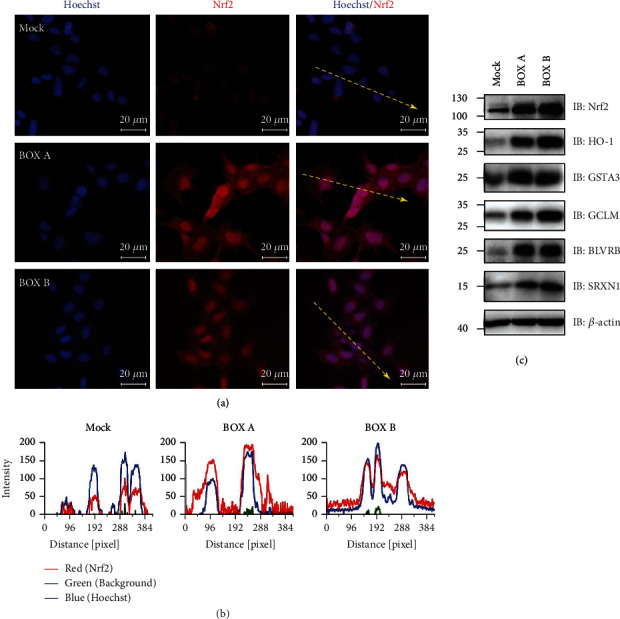
BOX A and BOX B induce the nuclear accumulation of Nrf2 and promote the antioxidant responsive gene expression on the protein level. (a) Representative immunofluorescent staining of Nrf2 distribution and nuclear accumulation in SH-SY5Y cells. The cell treatment with BOX A or BOX B (30 *μ*M) for 24 h was stained with anti-Nrf2 antibody (red) and counterstained with Hoechst33342 (blue). Bar 20 *μ*m. (b) Intensity profile of different fluorescent staining as the yellow dashed line with arrow shown in (a) in pixel distance (left, Mock; middle, BOX A; right, BOX B). The intensity profile of the line was measured by profile of the ZEN 2.6 lite software (Carl-Zeiss). Red, Nrf2 staining; blue, Hoechst33342; green, background noise. (c) Western blot showing the protein expression of Nrf2 and downstream target gene expression in 11 DIV cultured PCNs treated with BOX A or BOX B (30 *μ*M each) for 48 h. One representative blot of 3-4 independent experiments.

To further characterize the molecular pathway, Nrf2 immunofluorescence staining was employed to detect the Nrf2 translocation in human neuroblastoma cell SH-SY5Y after BOXes administration. The immunostaining results showed that Nrf2 accumulated in the nucleus of the BOX A or BOX B-treated cells but not the control cells (Figures 6(a) and 6(b)). In addition, immunoblotting analysis showed that the protein expression of downstream signals of Nrf2 including HO-1, GCLM, BLVRB, GSTA3, and SRXN1 was also markedly increased (Figure 6(c)).

## 4. Discussion

The cellular mechanisms underlying SAH are diverse, including oxidative stress, inflammation, brain edema, and apoptotic neuronal cell death [3]. One major factor, however, is the red blood cell lysis, the release of heme, and the subsequent degradation cascade, which collectively have a strong impact on the affected neurons and their electrical excitability [5–7]. A series of HHDPs have been recently identified, and the BOXes were found to be clinically associated with the progression of DIND after SAH [8, 9, 18], but their direct roles in neurons remain largely unknown. In this study, we synthesized BOX A and BOX B according to the protocols reported previously [14, 19] and observed their impacts on primary neurons and neuronal cell lines. The findings include an increase of ROS production and depolarized mitochondrial membrane potentials triggered by BOXes, which activate the Nrf2 signaling pathway to mediate the antioxidant response in cortical neurons and neuroblastoma cells.

To surmount, a total synthesis method with small modifications (Figure 1(a)) was employed to the bottom-up synthesis of BOX A and BOX B from bromocitraconic anhydride and obtained high-purity BOXes (>97%). Furthermore, the pharmacokinetics studies of synthesized BOXes showed a very high concentration distribution between brain and peripheral blood, and BOX B had a much larger B/P ratio than BOX A 30 min after *in vein* injections (Figures 1(b) and 1(c)), which directly demonstrates that BOXes are transported across the blood-brain barrier.

There is accumulated evidence that BOXes are closely related to the pathophysiology of DIND after SAH [8, 9]. Seidel et al. used a very high dose of BOXes (>500 *μ*M) and found a change in the morphology and the redox potential (100 *μ*M) of hepatocytes [12]. Here we explored the direct role of BOXes in isolated cortical neurons and found that a moderate concentration of BOXes (10-30 *μ*M) induces ROS production (Figure 3) without obvious alterations in cell viability and apoptosis of neurons (Figure 2), which is consistent with the previous report that a moderate concentration (~25 *μ*M) of BOX A or BOX B did not affect SH-SY5Y cell viability [24]. Of note, the mitochondrial potential was decreased in a dose-dependent manner after treatment with BOX A or BOX B (Figure 4). The impact of BOXes on ROS generation may advance a concept that BOXes trigger oxidative stress and affect the neurons and eventually lead to DIND after SAH. This is consistent with previous findings that BOXes alter the redox potential in hepatocellular cells [12] and oxidative stress as well as BOXes significantly increased after intracerebral hemorrhage [4]. Yet, the underlying molecular mechanisms are possibly different and require further studies.

**Figure 7:**
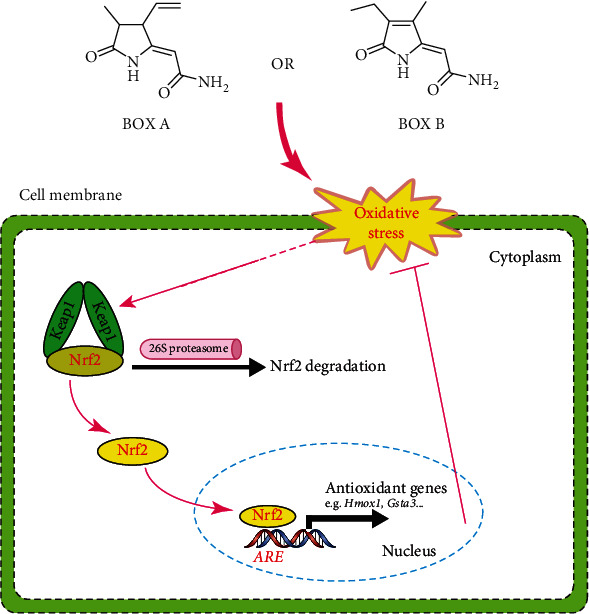
Proposed model of BOX A or BOX B inducing oxidative stress and activation of antioxidant response in neurons. BOX A or BOX B induced oxidative stress and enhanced the nuclear accumulation of Nrf2 to activate the transcription of target genes harboring antioxidant response element (ARE).

Redox homeostasis is tightly regulated by many endogenous redox systems, the cellular antioxidant response mechanisms, or absorption of an antioxidant substance from outside [25]. In neurons and many other cell types, Nrf2 is a master regulator of redox sensors, kept at very low levels *via* complexing with Keap1 and to be subjected to proteasome-mediated degradation at basal conditions. Activation of Nrf2 leads to translocation to the nucleus to initiate antioxidative response gene transcriptions and phase I/II enzyme expression [26, 27]. In addition, the Nrf2 pathway has been shown to be involved in bilirubin-mediated oxidative stress [28]. In the present study, the bilirubin metabolites BOX A and BOX B triggered the oxidative stress and induced several oxidative stress-responsive genes, including *Hmox1*, *Blvrb*, *Gclm*, *Srxn1*, and *Gsta3*. This induction is likely due to Nrf2 activation and translocation to nucleus (Figure 6) to activate the antioxidant response element (ARE) transcriptional cascade (Figure 5) [29]. The ARE-regulated genes including *Hmox1*, *Blvrb*, *Gclm*, *Srxn1*, and *Gsta3* are extensively investigated and contribute to oxidative stress [29–32]. Inducible *Hmox1* knockout mice have a smaller cerebral hemorrhagic lesion [33], which supports the toxicity of higher levels of BOXes that cause the upregulated *Hmox1* in cortical neurons from both mRNA and protein levels (Figures 5(b), 5(c), and 6(c)). Intriguingly, our findings showed that the Nrf2 accumulation and nuclear translocation and subsequent transcription cascade is triggered by BOXes, which to some extent, is contrary to a previous study that the Nrf2 knockout mice have an enlarged cerebral hemorrhagic lesion [34], indicating a cytoprotective feedback mechanism in BOXes treated neurons. Therefore, BOXes driving the oxidative stress and perturbing the redox homeostasis lowers mitochondrial membrane potential, which may trigger an antioxidative response pathway (Nrf2): Nrf2 is translocated to the nucleus to initiate antioxidative responsive gene transcription, to antagonize this process to maintain the attacked cell survival (Figure 7). Collectively, Nrf2 activation triggered by BOXes provides insight into the pathophysiological mechanism of DIND after SAH or other neurological dysfunctions related to cerebral hemorrhage.

## 5. Conclusion

Using synthesized high-purity BOXes and cultured primary cortical neurons, we firstly provide evidence that BOXes directly increase both oxidative and antioxidative signals in neurons. Their mitochondria membrane potential lowering effect, a well-established proapoptosis marker, strongly suggests that BOXes may compromise neuronal functions after SAH. Further research is required to understand the function of BOXes in the development and progression of DIND.

## Data Availability

The data used to support the findings of this study are available from the corresponding author upon request.

## Supplementary Materials

Supplementary Materials Supplemental Figure 1: mass spectra (m/z) of synthesized BOX A (upper panel) and BOX B (bottom panel). m/z ~179.28 and 179.23 show the peak of BOX A and BOX B, respectively. Supplemental Figure 2: GO enrichment of DEGs induced by the BOX A (a) or BOX B (b) in cortical neurons. The DEGs (∣Foldchange | >2.00) were selected to GO enrichment statistical analysis, and the top 10 of each molecular function, cellular component, and biological process were presented, *P* < 0.05. Supplemental Table 1: selected DEGs in PCN treated with BOX A or BOX B. ∣Foldchange | >2.00 in BOX A- or BOX B-treated PCNs were selected.
