# Supplementary material for: Bilirubin Oxidation End Products (BOXes) Induce Neuronal Oxidative Stress Involving the Nrf2 Pathway
Source: Oxid Med Cell Longev. 2021 Jul 30;2021:8869908. doi: 10.1155/2021/8869908 (PMC8349295; doi:10.1155/2021/8869908)
Supplement: Supplementary Materials — Supplemental Figure 1: mass spectra (m/z) of synthesized BOX A (upper panel) and BOX B (bottom panel). m/z ~179.28 and 179.23 show the peak of BOX A and BOX B, respectively. Supplemental Figure 2: GO enrichment of DEGs induced by the BOX A (a) or BOX B (b) in cortical neurons. The DEGs (∣Foldchange | >2.00) were selected to GO enrichment statistical analysis, and the top 10 of each molecular function, cellular component, and biological process were presented, P < 0.05. Supplemental Table 1: selected DEGs in PCN treated with BOX A or BOX B. ∣Foldchange | >2.00 in BOX A- or BOX B-treated PCNs were selected. [file 8869908.f1.docx]

**
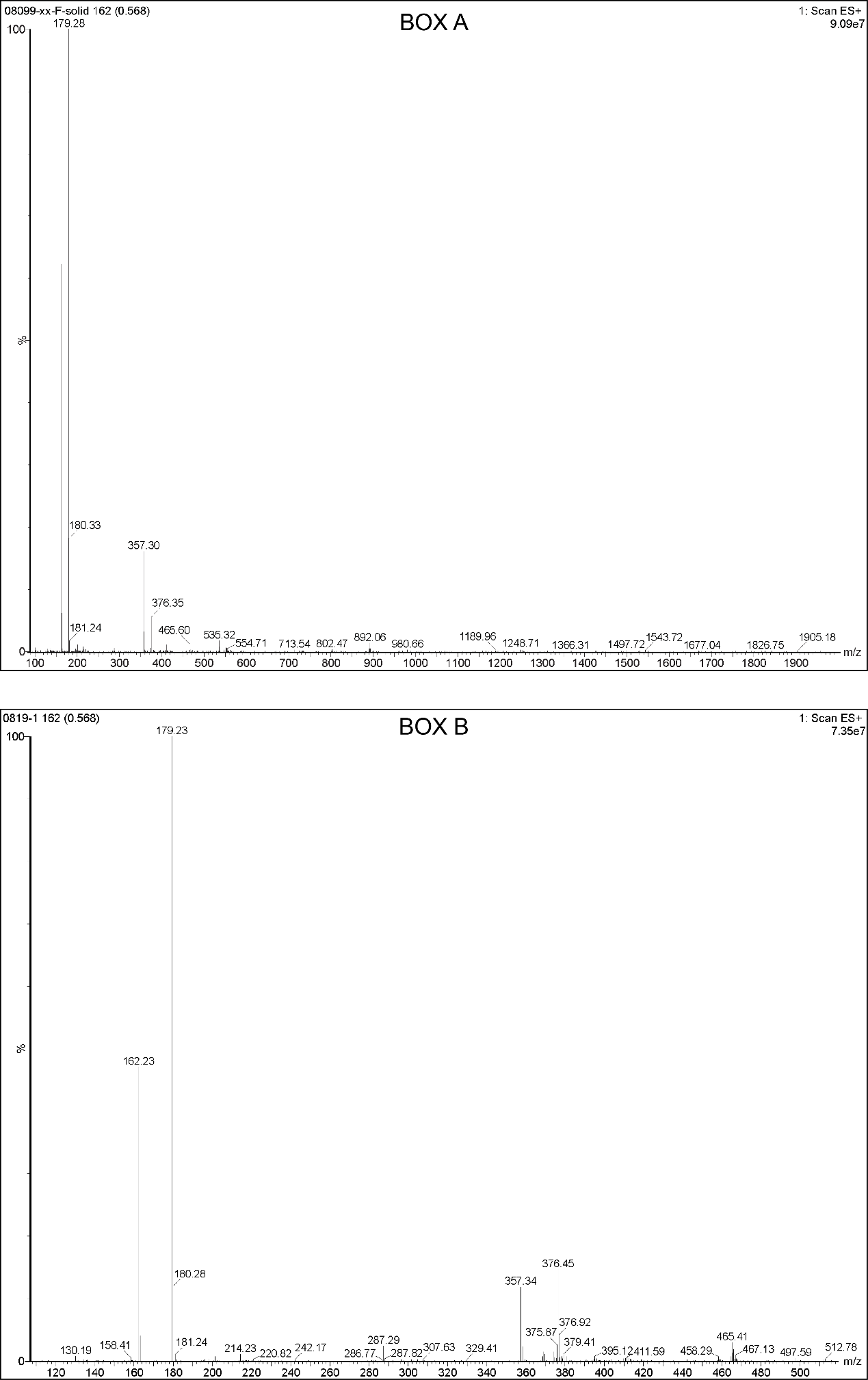
**

**Supplemental Figure 1.** LC-MS identified the synthesized BOX A and BOX B

**
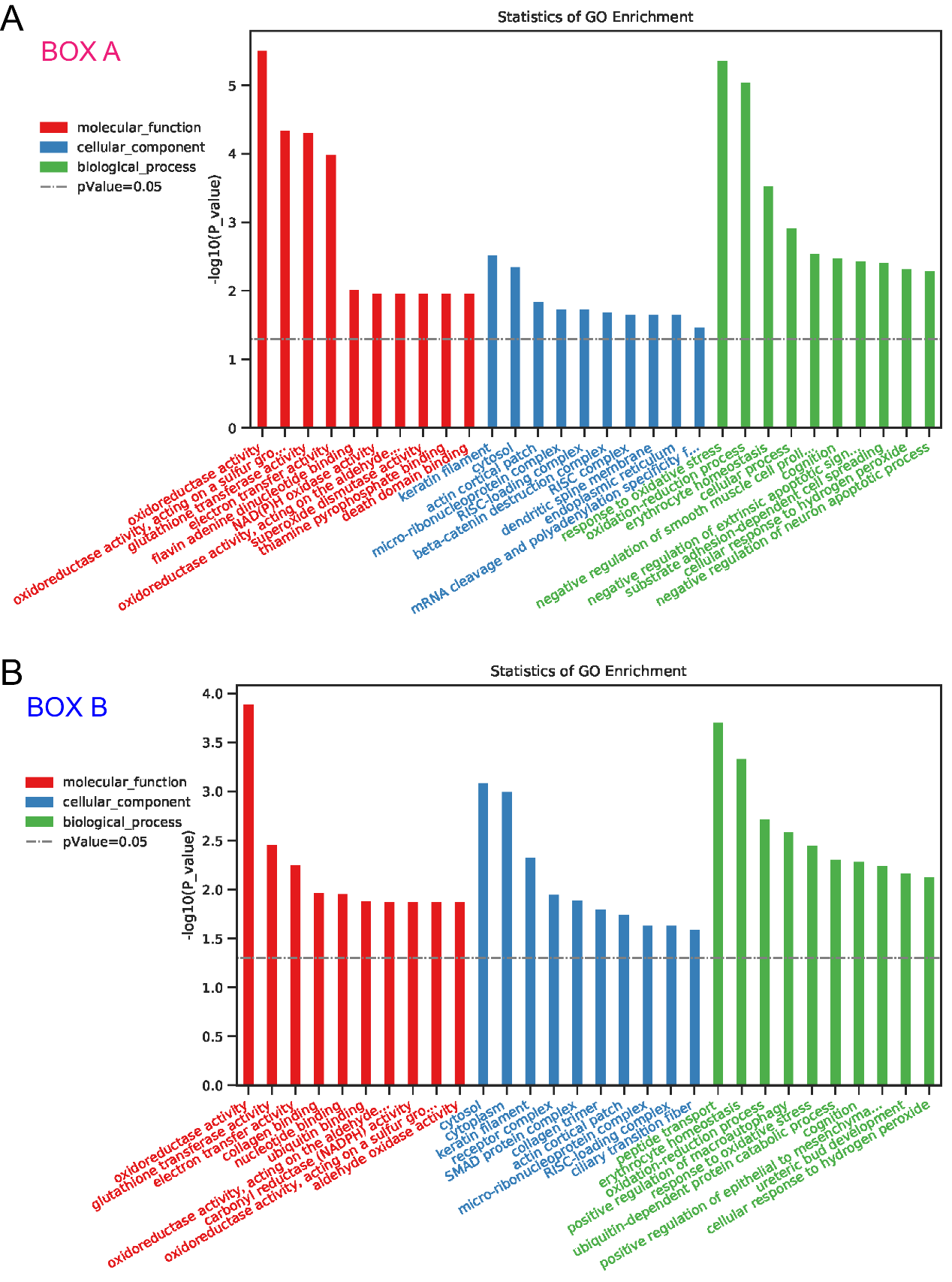
**

**Supplemental Figure 2.** GO enrichment of DEGs induced by the BOX A or BOX B in cortical neurons.

| **Supplemental Table 1 Selected DEGs in PCN treated with BOX A or BOX B** | | | |
| --- | --- | --- | --- |
| **GeneSymbol** | **Foldchange (BOX A)** | **Foldchange (BOX B)** | **GeneName** |
| Ago4 | 0.15 | 0.20 | argonaute RISC catalytic subunit 4 |
| Akr1c14 | 1.90 | 2.24 | aldo-keto reductase family 1, member C14 |
| Anks6 | 0.04 | 0.22 | ankyrin repeat and sterile alpha motif domain containing 6 |
| Aox1 | 2.19 | 2.01 | aldehyde oxidase 1 |
| Ar | 2.06 | 1.70 | androgen receptor |
| Axl | 0.33 | 0.35 | AXL receptor tyrosine kinase |
| Blvrb | 2.80 | 2.27 | biliverdin reductase B (flavin reductase (NADPH)) |
| Btbd10 | 1.05 | 2.09 | BTB (POZ) domain containing 10 |
| Btbd35f14 | 0.02 | 0.02 | BTB domain containing 35, family member 14 |
| Cacng2 | 2.69 | 0.82 | calcium channel, voltage-dependent, gamma subunit 2 |
| Cbr3 | 2.44 | 2.04 | carbonyl reductase 3 |
| Cntnap5b | 0.60 | 13.75 | contactin associated protein-like 5B |
| Col1a1 | 0.76 | 0.38 | collagen, type I, alpha 1 |
| Col7a1 | 1.08 | 2.62 | collagen, type VII, alpha 1 |
| Csnk1a1 | 2.93 | 3.37 | casein kinase 1, alpha 1 |
| Cul9 | 1.14 | 6.54 | cullin 9 |
| D5Ertd615e | 1.05 | 42.93 | DNA segment, Chr 5, ERATO Doi 615, expressed |
| Dapl1 | 0.45 | 0.63 | death associated protein-like 1 |
| Dcn | 0.80 | 0.39 | decorin |
| Dzip1 | 1.02 | 3.36 | DAZ interacting protein 1 |
| Esp23 | 0.02 | 0.07 | exocrine gland secreted peptide 23 |
| Fam221b | 0.72 | 13.97 | family with sequence similarity 221, member B |
| Gclm | 2.46 | 1.97 | glutamate-cysteine ligase, modifier subunit |
| Gm10639 | 6.42 | 3.09 | predicted gene 10639 |
| Gm14005 | 2.43 | 2.44 | predicted gene 14005 |
| Gm805 | 0.02 | 0.03 | predicted gene 805 |
| Gmeb1 | 0.98 | 0.34 | glucocorticoid modulatory element binding protein 1 |
| Gmppa | 1.25 | 0.50 | GDP-mannose pyrophosphorylase A |
| Grcc10 | 1.18 | 0.45 | gene rich cluster, C10 gene |
| Gsta2 | 3.81 | 1.94 | glutathione S-transferase, alpha 2 (Yc2) |
| Gsta3 | 8.79 | 4.57 | glutathione S-transferase, alpha 3 |
| H19 | 0.50 | 0.41 | H19, imprinted maternally expressed transcript |
| Hdgf | 0.23 | 0.86 | hepatoma-derived growth factor |
| Hmox1 | 2.28 | 2.24 | heme oxygenase 1 |
| Ilvbl | 0.50 | 1.01 | ilvB (bacterial acetolactate synthase)-like |
| Itga8 | 2.44 | 1.64 | integrin alpha 8 |
| Itgb4 | 1.02 | 67.04 | integrin beta 4 |
| Katnal2 | 0.85 | 0.15 | katanin p60 subunit A-like 2 |
| Kbtbd8 | 1.99 | 0.42 | kelch repeat and BTB (POZ) domain containing 8 |
| Krt13 | 0.01 | 0.01 | keratin 13 |
| Lgr4 | 10.95 | 0.97 | leucine-rich repeat-containing G protein-coupled receptor 4 |
| LOC108167801 | 2.20 | 0.88 | uncharacterized LOC108167801 |
| Mfsd2a | 2.31 | 2.88 | major facilitator superfamily domain containing 2A |
| Mocos | 2.05 | 1.72 | molybdenum cofactor sulfurase |
| Nkpd1 | 0.81 | 8.04 | NTPase, KAP family P-loop domain containing 1 |
| Nqo1 | 3.54 | 2.49 | NAD(P)H dehydrogenase, quinone 1 |
| Olfr774 | 1.02 | 55.27 | olfactory receptor 774 |
| Osgin1 | 2.04 | 2.10 | oxidative stress induced growth inhibitor 1 |
| Pck2 | 0.88 | 2.45 | phosphoenolpyruvate carboxykinase 2 (mitochondrial) |
| Pctp | 0.99 | 14.07 | phosphatidylcholine transfer protein |
| Pde6h | 2.82 | 0.90 | phosphodiesterase 6H, cGMP-specific, cone, gamma |
| Pgd | 2.02 | 1.37 | phosphogluconate dehydrogenase |
| Pir | 2.24 | 2.17 | pirin |
| Pla2g2e | 0.02 | 0.02 | phospholipase A2, group IIE |
| Ptcd1 | 1.03 | 7.52 | pentatricopeptide repeat domain 1 |
| Ptchd1 | 2.09 | 1.04 | patched domain containing 1 |
| Rida | 2.05 | 1.49 | reactive intermediate imine deaminase A homolog |
| Rnf43 | 0.20 | 0.23 | ring finger protein 43 |
| Serpina1d | 0.45 | 22.96 | serine (or cysteine) peptidase inhibitor, clade A, member 1D |
| Slc15a4 | 1.68 | 31.57 | solute carrier family 15, member 4 |
| Slc26a6 | 4.00 | 0.97 | solute carrier family 26, member 6 |
| Slc7a11 | 2.50 | 1.50 | solute carrier family 7 (cationic amino acid transporter, y+ system), member 11 |
| Slc9a9 | 1.07 | 9.63 | solute carrier family 9 (sodium/hydrogen exchanger), member 9 |
| Sly | 1.01 | 67.78 | Sycp3 like Y-linked |
| Smad3 | 0.92 | 2.08 | SMAD family member 3 |
| Spata9 | 0.30 | 0.25 | spermatogenesis associated 9 |
| Srxn1 | 3.06 | 2.56 | sulfiredoxin 1 homolog (S. cerevisiae) |
| Syn3 | 0.75 | 5.40 | synapsin III |
| Tap2 | 1.02 | 63.98 | transporter 2, ATP-binding cassette, sub-family B (MDR/TAP) |
| Tmem202 | 1.18 | 3.58 | transmembrane protein 202 |
| Tubb1 | 0.77 | 10.60 | tubulin, beta 1 class VI |
| Txnrd1 | 2.51 | 1.81 | thioredoxin reductase 1 |
| Ube2l6 | 0.89 | 2.76 | ubiquitin-conjugating enzyme E2L 6 |
| Ugt1a6b | 2.17 | 1.85 | UDP glucuronosyltransferase 1 family, polypeptide A6B |
| Usp48 | 0.95 | 3.53 | ubiquitin specific peptidase 48 |
| Vipr2 | 2.03 | 1.01 | vasoactive intestinal peptide receptor 2 |
| Wipf1 | 0.40 | 0.48 | WAS/WASL interacting protein family, member 1 |
